# Rethinking Model Transferability: Validity Domains as a New Approach to Delineate the Limits of Bloom Date Projections

**DOI:** 10.1111/gcb.70776

**Published:** 2026-03-11

**Authors:** Julian N. Bauer, Katja Schiffers, Lars Caspersen, Hisayo Yamane, Eike Luedeling

**Affiliations:** ^1^ Institute of Crop Science and Resource Conservation (INRES) University of Bonn Bonn Germany; ^2^ Graduate School of Agriculture Kyoto University Kyoto Japan

**Keywords:** cherry blossom, climate scenarios, machine learning, model extrapolation, phenology, process‐based

## Abstract

Accurately predicting future events under novel environmental conditions is a central challenge in modeling, especially when no validation data are available. While model transferability is often discussed through the concept of a “forecast horizon,” we expand this framework by introducing the concept of “validity domains.” These consider not only the extrapolation distance from the calibration data but also the absolute position of calibration and application conditions along an environmental gradient. Using phenological observations from Japanese Yoshino cherry (*Prunus* × *yedoensis*) across a climate gradient in Japan, we calibrated process‐based and machine learning models for each of 48 locations and validated them with data from all other locations. Interpolating model performance metrics yielded a continuous synthetic surface of predictive accuracy across the full observed temperature range, from which we delineated model‐specific validity domains and assessed how transferability depends on both model type and calibration environment. Our findings show that process‐based models retain broader validity when calibrated in colder environments but degrade in warmer settings. In contrast, machine learning models exhibit narrower but more consistent validity across the gradient. These systematic differences reveal that the location of calibration and the structure of the model fundamentally shape its reliability under new conditions. By identifying where prediction errors remain below a context‐specific validity threshold, our approach provides a robust framework for assessing model applicability under shifting climate conditions. Mapping validity domains offers practical guidance for model selection and allows quantifying how far models can be pushed before their predictions become unreliable.

## Graphical abstract

Accurately predicting future phenology events in novel environmental conditions is a key challenge in modelling. Using first bloom observations of the Japanese Yoshino cherry across a temperature gradient, we calibrated process‐based and machine learning models at multiple locations and cross‐validated them to obtain the predictive performance for all combinations. By interpolating these outcomes, we derived continuous performance surfaces that reveal how model reliability changes along the gradient. Our analysis shows that process‐based models retain broader validity when calibrated in colder environments, whereas machine learning models exhibit more consistent but narrower validity. These validity domains demonstrate that both model structure and the calibration environment fundamentally influence transferability, offering a framework for identifying where predictions remain reliable under changing climate conditions.

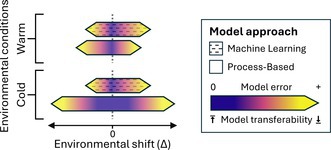

## 1 Introduction

To anticipate the impacts of climate change, decision‐makers and researchers rely heavily on simulation models. In environmental sciences, models are frequently used to predict future ecosystem functioning (Scholze et al. 2006), species distributions (Araújo et al. 2005; Nolzen et al. 2022), agricultural yields (Lobell and Burke 2010) and phenological processes (Ibáñez et al. 2010; Primack et al. 2009). Such simulation models are typically calibrated using observed historical or recent data collected from a range of environmental conditions. The process of calibration describes the search for suitable model parameters that align the model's predictions with available observations. To evaluate the results of the calibration, models are validated using independent observations that were not part of the calibration process. These observations are typically collected under conditions similar to those used for calibration. However, validating a model under similar conditions does not ensure robust predictive performance under different or novel scenarios, such as those imposed by global warming (Asse et al. 2020; Luedeling et al. 2021).

Aiming to gauge model transferability, many studies have tested to what extent models can be applied to conditions beyond those of the calibration data within spatial, temporal, or phylogenetic dimensions (Bell and Schlaepfer 2016; Sequeira et al. 2018; Thuiller et al. 2004; Yates et al. 2018). Yet due to lack of data, model transferability is often quantified only for a few individual conditions, leaving differences and underlying relations in model performance undetected. To provide a more nuanced assessment, we present a new way of revealing continuous transferability patterns along a broad range of conditions.

**FIGURE 1:**
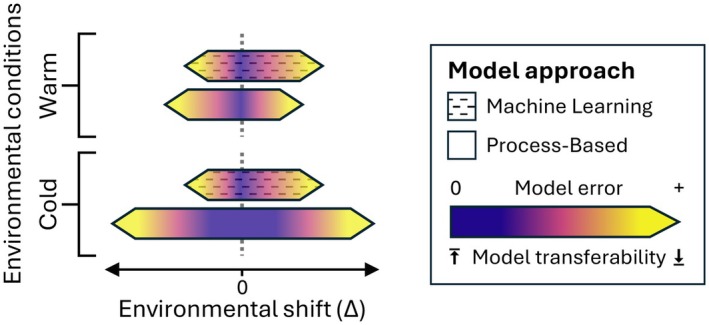
Visualization of the validity domain concept for two model approaches under two calibration conditions. The width of the domains and the model error, indicated by shape and gradient, depend on the environmental shift (Δ = *T*_valid_—*T*_calib_).

Studies on model transferability often refer to the concept of a “forecast horizon,” which was introduced by Petchey et al. (2015). The main purpose of this framework is to appreciate how far into the future—or how far along an environmental gradient—a model can safely be used before its predictions become unreliable. We argue that the distance to these forecast horizons is not only a property of each specific model but also depends on the absolute position along the environmental gradient at which the model was calibrated. Especially for systems that display non‐linearities or tipping points that are not adequately captured by a model's structure, forecast horizons may be very short when approaching the conditions where these non‐linearities occur. We introduce the concept of “validity domains” (Figure 1) to expand on the “forecast horizon” framework by focusing on both the extrapolation distance from the calibration data (in terms of environmental conditions) and the absolute position of calibration and extrapolation conditions along the environmental gradient. The validity threshold—defined as the minimal acceptable prediction accuracy—needs to be determined in a case‐ and context‐specific manner (Yates et al. 2018) and is equivalent to the “forecast proficiency threshold” proposed by Petchey et al. (2015). For calibration‐validation combinations within these validity domains, model transferability is given, with prediction errors typically below the threshold. Mapping model validity domains helps to validate models for application in particular climatic settings. It also allows quantifying the time horizon for which models can be expected to remain reliable in a changing climate.

To test models for transferability and illustrate the concept of validity domains, we used the example of a publicly available long‐term dataset on the timing of Yoshino cherry blossom (*Prunus* × *yedoensis*) in Japan. Flowering in cherry trees, as in many other temperate‐zone trees, is mediated by temperature conditions during the preceding winter and spring. Such trees spend the winter in a state of dormancy, during which growth is suspended to reduce the trees' vulnerability to low temperatures. To overcome dormancy, tree buds need to experience a sequence of cool and warm temperatures (Samish 1954; Saure 1985). The dormancy period is therefore usually conceptualized as a two‐stage process. During endodormancy, trees accumulate chill until phase‐specific requirements are met (Lang et al. 1987). They then enter the ecodormancy phase, during which trees can register exposure to heat. When heat requirements are fulfilled, trees resume their growth. Changes in temperature during dormancy, such as the recent warming resulting from climate change, can affect the timing of phenological stages. In most cases reported in the literature, warming during winter has resulted in earlier occurrence of spring events (Menzel et al. 2006; Parmesan and Yohe 2003) and in many species shifting their ranges towards the poles (Parmesan and Yohe 2003). However, some species have also responded with delays in the timing of spring phases. This has been particularly noticeable for temperate‐zone trees growing in warm‐winter climates with low chill availability. Specific examples of such responses include delayed apricot bloom in low‐chill regions of China (Guo et al. 2015) and delayed cherry blossom in southern Japan (Hsu et al. 2023).

Different types of modeling approaches are available to predict the time of flowering. Process‐oriented models express underlying biological concepts (e.g., endo‐ and ecodormancy) as mathematical equations. The parameters of these models are calibrated to increase accuracy and adapt the model to the actual application data. Machine‐learning models form a different approach, where algorithms search for the best fit between independent and dependent variables. In this way, underlying processes are mostly neglected and structures in the data are prioritized. A key advantage of machine‐learning models is their ability to adapt seamlessly to various use cases without requiring prior, case‐specific adjustments or customization. This advantage made machine‐learning approaches popular for model transferability studies, especially in the field of species distribution modeling (Guillera‐Arroita et al. 2015; Petchey et al. 2015; Yates et al. 2018). Some studies have compared process‐based models with purely statistical models (Higgins et al. 2020), while others have highlighted the lack of model transferability studies for process‐based approaches (Yates et al. 2018). In previous phenology modeling studies, process‐oriented models usually provided more reliable predictions under conditions outside the calibration range compared to machine‐learning models (Luedeling et al. 2021; Medlyn et al. 2011; Piao et al. 2019).

The main contribution of this study is to introduce the validity domain concept to systematically uncover changes in performance for phenology models calibrated along a continuous temperature gradient. To achieve this, we extend the forecast horizon concept to refine assessments of model transferability. We applied this method to a process‐based and a machine‐learning model. Validity domains can be particularly insightful when application conditions are prone to change. We find that narrowly calibrated models transferred to future climate scenarios are often affected by considerable errors. With this study, we want to raise awareness of the pitfalls of using models for extrapolation by visualizing reductions in accuracy for a broad range of conditions.

## 2 Materials and Methods

We accessed data of first bloom phenology for Yoshino cherries (*Prunus* × *yedoensis*) through the website of the Japan Meteorological Agency (Japanese Meteorological Agency 2023b). The dataset contained records for 102 locations, of which we selected 48 for further analysis. All the selected locations have a weather station within a radius of < 5 km, with most of the specimen trees located very close to the stations. The Japan Meteorological Agency operates these weather stations (Japanese Meteorological Agency 2023a), but the data are also stored in the Global Summary of the Day database (NOAA National Centers of Environmental Information 1999) from where we accessed it. For every location, we selected all years with phenological observations missing < 6 days of daily minimum and maximum temperatures during the period that is relevant for bloom prediction (previous September to May of the year of recorded bloom). A detailed table summarizing the available years for each location is provided in Table S1. Across these locations, seasonal mean temperatures range from 5.1°C in Sapporo to 15.4°C in Kagoshima. The distribution of locations along this gradient is generally balanced, with two more densely represented clusters near 11.5°C and 13.6°C. Across sites, data availability ranged from 27 to 64 years, with an average of approximately 46 years. We interpolated the remaining gaps in the daily data. Since the phenology models we used require hourly temperature data as inputs, we constructed hourly data from daily minimum and maximum temperature. This conversion was based on a sine function for the temperature development during the day and a logarithmic decay function for the night, with location‐specific sunrise and sunset times computed from the locations' latitudes. For the application in the original study, this interpolation method resulted in an RMSE of 2.5°C (Luedeling 2018).

To model tree phenology in a process‐based manner, we used the PhenoFlex framework developed by Luedeling et al. (2021). PhenoFlex consists of two sub‐models, the Dynamic Model (Fishman et al. 1987b, 1987a) for chill accumulation and the Growing Degree Hours Model (Anderson et al. 1986) for heat. The framework allows for a gradual transition between the two major dormancy phases (endo‐ and ecodormancy). We fitted the 12 model parameters as recommended in the original publications (Luedeling et al. 2021; Urbach et al. 2024). We calibrated models for each of the selected locations and then systematically evaluated the models with observed phenology data from all other locations. All 48 parameter sets were thus tested on all 47 remaining datasets from the other locations as well as with the original calibration data, resulting in 2304 calibration‐validation combinations. To characterize validity domains for a machine‐learning model, we used the Gaussian process model (Rasmussen and Williams 2005; Williams and Barber 1998) included in the kernlab R package (Karatzoglou et al. 2024) to calibrate the models, following the same procedure as for the PhenoFlex framework. After calibration, we systematically applied the models to all other validation datasets.

We used the root mean square error (RMSE) to evaluate model performance for both approaches. For some years in certain combinations of calibration and validation datasets, the PhenoFlex framework was unable to predict bloom dates. To adequately represent these prediction failures, we set all non‐computable values to an arbitrary error of 35 days. All combinations were successfully computed by the Gaussian process model. In the next step, we applied the Kriging method (Cressie 1993) to interpolate the results of the individual combinations into a continuous two‐dimensional surface. On this surface, we subsequently delineated validity domains using an RMSE threshold of 5 days for both approaches. For the process‐based approach, we interpolated RMSE values along the identity diagonal, where each model was evaluated on the same location used for calibration. For the machine‐learning approach, RMSE values on the identity diagonal were set to zero, reflecting the assumption that a Gaussian Process model can achieve an essentially perfect fit when trained and tested on datasets of this size. These diagonal values were interpolated prior to the Kriging step to ensure that the spatial smoothing procedure did not distort or dominate valid performance patterns across locations, especially at the warmer end of the temperature range where data points become scarcer.

To evaluate model performance for different climate scenarios, we matched future projections to similar temperature conditions contained in the historically observed bloom records. To this end, we simulated future weather for two time windows: 2035–2065 and 2070–2100. The medians of these periods are the scenario years 2050 and 2085, for which the simulations can be considered representative. For each of the time slices, we created scenarios for the Shared Socioeconomic Pathways (SSPs) SSP1.26, SSP2.45 and SSP5.85 (IPCC 2023). We extracted all available data for all climate models from the CMIP6 (Eyring et al. 2016) stored in the Copernicus Climate Data Store maintained by the European Centre for Medium‐Range Weather Forecasts (Copernicus Climate Change Service 2021). A full list of all GCMs analyzed in this study can be found in Table S2. For each combination of year, SSP and climate model, we created 100 years of synthetic weather using the RMAWGEN weather generator (Cordano and Eccel 2019). The reference period for our simulation was the time period between 1986 and 2014, with a median of 2000 as the reference year. This reference period covers a climatologically representative period that describes the average climatic conditions of each location and serves as a baseline for the relative changes introduced by the scenarios. After generating weather data, we calculated the mean temperature for the period between September and June of the following year for each of the years and all climate models. We then averaged these mean values for each combination of year and SSP. The practice of using the mean as a proxy for overall seasonal characteristics conceals the phase‐specific temperatures during winter and spring, which may differ for two seasons with identical mean values. Across historical and future data, computed correlations indicate a closer connection between seasonal mean temperature and winter conditions (*r* = 0.8) than with spring conditions (*r* = 0.6). The complete process of obtaining, preparing and analyzing the data, except for the download of the phenology data and the Gaussian process model, was carried out using the chillR package (Luedeling et al. 2023) in R (R Core Team 2023). The model calibration and the simulation of the weather for the future scenarios were carried out on the massively parallel processor cluster “Bonna” of the University of Bonn.

## 3 Results

**FIGURE 2:**
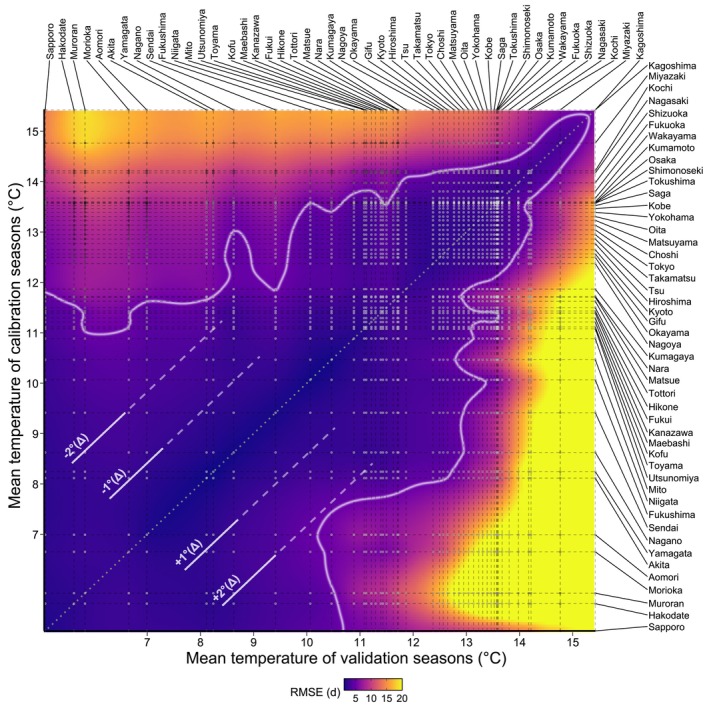
Performance of the process‐based approach for all calibrated models and validation data sets. We created models and selected validation data based on temperature and cherry blossom phenology records from 48 Japanese locations (names on right and top axes). Horizontal lines mark the seasonal (September–May) mean temperature of the data used for model calibration at each location, and vertical lines mark the mean temperature of the validation data at each location. Each line intersection represents an actual calculated Root Mean Square Error (RMSE) value based on a calibrated model from one site and validation data from another site. All other points are interpolated from this initial grid of combinations using Kriging. The central grey dashed line marks the identity line, where calibration and validation data originate from the same location. RMSE values for these combinations were interpolated based on the original calibration performances along the diagonal. All RMSE values above 20 days are shown in yellow. RMSE values above 5 days are enclosed by a white line, which delineates the validity domains (minor exceptions within and outside this general area are omitted for clarity). The white (partly dashed) lines parallel to the central grey dotted line, indicate mean temperature shifts relative to the calibration data.

**FIGURE 3:**
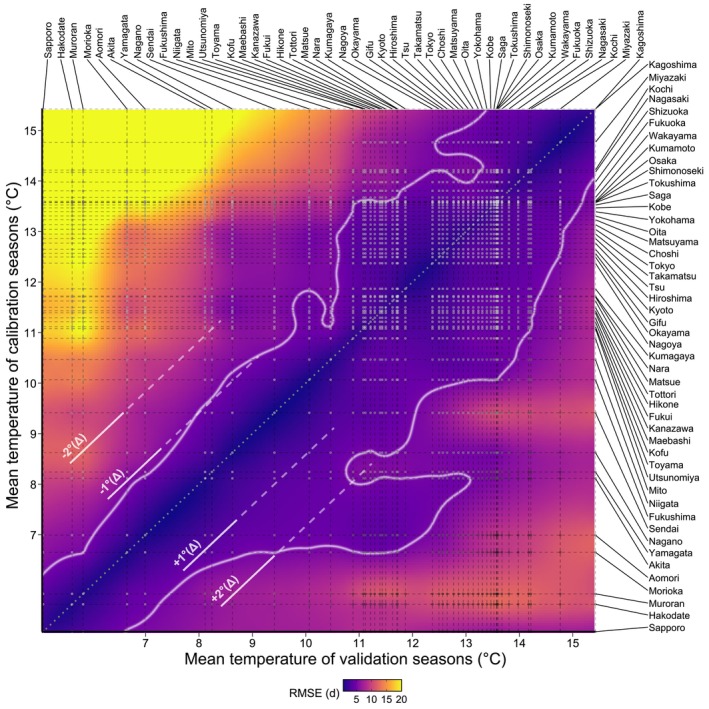
Performance of the machine‐learning approach for all calibrated models and validation data sets. We created models and selected validation data based on temperature and cherry blossom phenology records from 48 Japanese locations (names on right and top axes). Horizontal lines mark the seasonal (September–May) mean temperature of the data used for model calibration at each location, and vertical lines mark the mean temperature of the validation data at each location. Each line intersection represents an actual calculated Root Mean Square Error (RMSE) value based on a calibrated model from one site and validation data from another site. All other points are interpolated from this initial grid of combinations using Kriging. The central grey dotted line marks the identity line, where calibration and validation data originate from the same location. RMSE values for these combinations were manually set to 0. All RMSE values above 20 days are shown as yellow. RMSE values above 5 days are enclosed by the white line, which delineates the validity domains (minor exceptions within and outside this general area are omitted for clarity). The white (partly dashed) lines parallel to the central grey dotted line, indicate mean temperature shifts relative to the calibration data.

We assessed the transferability of the process‐oriented model (Figure 2) and the machine‐learning model (Figure 3) across the available temperature gradient by applying the models, calibrated under all conditions, to validation data representing all conditions. We observed that both approaches perform best when calibration and validation conditions are similar (close to the dotted identity lines in Figures 2 and 3). The combinations where model and validation data originated from the same location generally showed the highest performance. In general, performance declined with increasing differences between calibration and validation conditions. In the case of the process‐oriented model, we observed consistently high performance among cold‐climate locations (6°C–12°C), where deviation from calibration temperature conditions reduced the performance only slightly. With increasing mean temperature (12°C–15°C), the range of calibration‐validation combinations that result in good performance becomes narrower. In this temperature range, model transferability is only given when the underlying calibration and validation conditions match moderately well. Under high‐temperature conditions (15°C–16°C), only models calibrated under very similar conditions demonstrated reasonable performance, but even then, prediction accuracy was relatively low, with RMSE values exceeding 5 days. Overall, model performance generally decreased with increasing differences between calibration and validation conditions, with the extent of this decline depending strongly on the temperature range, especially for the process‐based approach. Off‐diagonal tests revealed asymmetric degradation, with warm‐calibrated models displaying substantial errors when validated under cold conditions (~15 days RMSE) and cold‐calibrated models showing even larger errors when applied to warm validation conditions (> 20 days RMSE). In the latter case, a considerable number of applications resulted in failed predictions, especially when models calibrated under cold locations were applied to temperate or warm validation sites (see Figure S1 for the number of failed predictions across all combinations).

The machine‐learning models performed almost perfectly when they were validated in the same locations that were used for model calibration. However, this high performance declined sharply when the validation setting differed only slightly from the calibration conditions. For such conditions, errors were still below the validity threshold (RMSE < 5 days) but higher than when the process‐based approach was applied to the same site combinations. Many machine‐learning models calibrated in warm locations exhibited errors > 20 days when they were validated with data from cool climates. In contrast, models calibrated with data from cold locations performed reasonably well under a broad range of conditions, with errors in the range of 5–12 days. The decline in performance was uniform and less steep than for the process‐oriented model. Machine‐learning models showed an opposite off‐diagonal asymmetry, with warm‐calibrated models producing large errors under cold validation conditions (> 20 days RMSE), whereas cold‐calibrated models applied to warm validations showed only moderate errors (~15 days RMSE). In a direct comparison, the process‐oriented approach proved superior to the machine‐learning approach for cold‐calibrated models and for similar calibration and validation temperatures due to its better performance and greater validity. Model transferability towards much warmer validation conditions was greater for the machine‐learning model than for the process‐based model, but still produced relatively large errors of up to 12 days.

Further experiments using the regime‐clustered groups and the pooled global dataset yielded patterns consistent with the main findings. As shown in Figure S2 (subplots A and B), cross‐validation among the temperature‐regime groups and the global pool showed a similar performance degradation under increasingly divergent conditions, similar to the observed patterns of the approaches shown in Figures 2 and 3. When transferring the group models to individual locations (and vice versa), the process‐based approach remained stable with an even change in performance across settings. In contrast, the machine‐learning approach exhibited higher variability, with performance fluctuating more strongly when the location and temperature regime groups were validated with each other.

**FIGURE 4:**
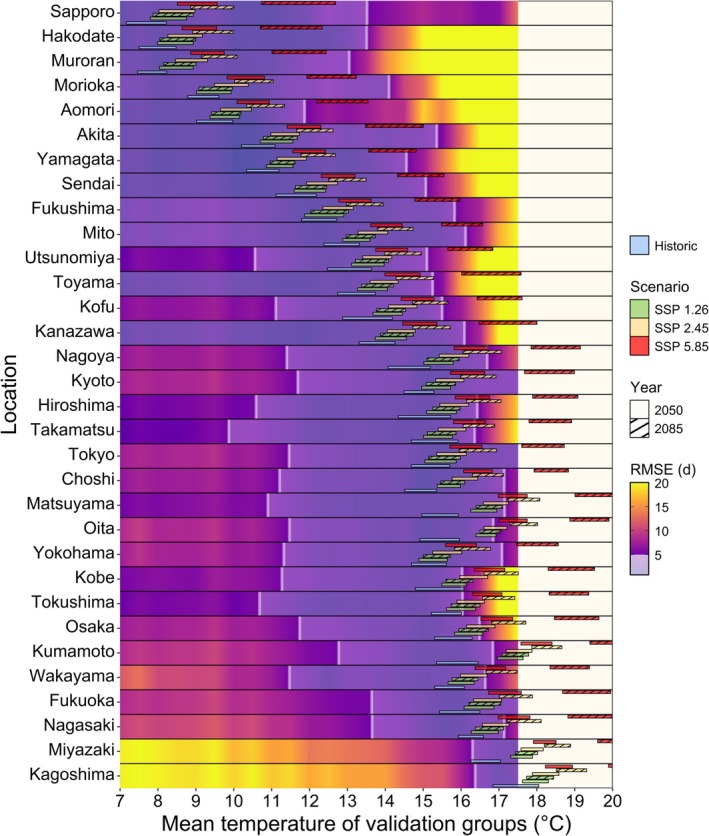
Distribution of simulated temperatures for future scenarios mapped onto the performance of 32 process‐based models from representative locations. Japanese cherry blossom phenology data and temperature records served as a basis for the model calibration and the assembling of 22 validation groups, each containing up to 30 thermally similar seasons from different locations. The coldest group contained temperature records for phenology seasons that had a mean temperature of 7.0°C–7.5°C, while the warmest group contained seasons with mean temperatures between 17.5°C and 18.0°C. We applied all selected models to all of these validation groups to generate an interpolated surface of model performance under various conditions used for a comparison with future scenarios. The simulations for the future scenarios are based on three shared socioeconomic pathways (SSPs), for each of which up to 19 global climate models (GCM) were available. For each of the SSPs and each of the available GCMs, we produced 100 simulations. The bars represent the interquartile range of the distribution of mean temperature during the phenology season across all 100 simulations and all GCMs. We used the root mean square error (RMSE) as performance metric. The white lines indicate the validity domains with an RMSE of 5 days. All errors greater than 20 days are displayed as yellow to avoid distortion of the color gradient. Available weather data recorded in the past at each location determined the historical distribution.

To gauge model transferability into future climates, we evaluated the performance of the process‐based model under validation conditions that corresponded to conditions projected by future climate scenarios. To this end, we created location‐independent validation datasets by grouping all observed seasons across all locations according to their seasonal temperature. This allowed us to apply models on groups of specific seasons with similar temperatures from various locations (e.g., location‐independent seasons with a mean temperature between 7.5°C and 8.0°C for the coldest group of validation seasons). Using these validation data and models from 19 representative locations, we calculated the performance of all newly combined calibration and validation conditions. Onto this, we overlayed future temperature distributions for the same representative locations obtained from weather simulations based on three shared socioeconomic pathways and 19 climate models from the CMIP6 model ensemble (Figure 4). This allowed us to anticipate the magnitude of errors that arise when using models calibrated with data from the past to project bloom under future temperature conditions at the same location. In general, we observed three patterns: (i) in cold locations, the expected errors remain small regardless of the climate scenario and scenario year, (ii) in moderately warm locations the expected errors remain small except for the pessimistic climate scenario SSP 5.85 by 2085, and (iii) in warm locations even bloom date projections for the most optimistic climate scenarios are prone to errors. The simulated weather conditions for the warmest locations exceeded the range of available validation data. In cold regions (from Sapporo to Aomori), we expect an RMSE ranging from 4 to 5 days under the moderate climate scenario SSP 2.45 by 2050 and even lower errors under the optimistic climate scenario SSP 1.26. For moderately warm locations (from Akita to Toyama), error rates remain similar to the previous locations, with RMSE peaks below 10 days. For the warmer locations (from Kanazawa to Nagasaki), applying models calibrated with observed data to project bloom under near‐future scenarios likely entails large errors. For such projections, even the most optimistic scenario SSP 1.26 is estimated to produce errors up to 15 days even in projections for 2050, the relatively near‐term future. From this we deduce that reliably projecting bloom dates for conditions that are even warmer than these relatively mild scenarios would face considerable challenges.

## 4 Discussion

We tested phenology model transferability by systematically calibrating models over a wide temperature gradient and assessing how the resulting models performed when used to make projections for different temperature regimes. Based on the results, we defined validity domains for each model to delineate the conditions for which a model can be considered reliable. For temperature conditions within these validity domains, we expect models to produce accurate phenology predictions.

The validity domains for the process‐based phenology models (Figure 2) center around the combinations where validation conditions match calibration conditions. Unsurprisingly, these combinations present the best fit of the models to the application data, since both originate from the same locations. We arranged the data by temperature and observed that model performance always declined with increasing difference from the calibration conditions, but that this decline varied strongly along the calibration temperature gradient. Models calibrated under cold conditions featured substantially wider validity domains than their warm‐location counterparts. A possible reason for this pattern may be that under warm conditions, bloom dates depend increasingly on chill accumulation during endodormancy, as opposed to heat during the subsequent ecodormancy phase (Guo et al. 2015). Since chill accumulation cannot be observed directly, chill models are difficult to develop and likely much less accurate than heat models, leading to greater prediction errors in chill‐limited than in heat‐limited settings. A further reason for poor model performance under warm conditions may be that under such conditions, dormancy dynamics do not fully adhere to the dormancy progression outlined by our process‐based model. Such poor performance under warm conditions has already been reported for experimental conditions (Fernandez et al. 2022), as well as in a study that used process‐based models on apple phenology data from various locations along a climatic gradient (Darbyshire et al. 2017).

Beyond these primary drivers, several additional factors may contribute to the observed degradation in warm‐calibrated performance. These include the shape of the chilling and forcing responses at high temperatures as well as the increasing influence of non‐temperature processes. Limited data availability at the warm end of the climatic gradient and potential local adaptation, which a single parametric formulation cannot capture, may further compound the degrading model performance and transferability.

It is also important to acknowledge that the Somei‐Yoshino cherry approaches its upper climatic limit in the warmest locations in the dataset. South of Kagoshima, this species is no longer commonly observed and is largely replaced by the more heat‐adapted *Prunus campanulata*. Such biological constraints, together with structural limitations of the model, may exacerbate prediction failures under warm conditions, particularly when transferring models calibrated in much colder environments to conditions located near the species' distributional limit.

Validity domains of the machine‐learning approach (Figure 3) differed markedly from those of the process‐based framework. For the machine‐learning model, the errors obtained for equal calibration and validation conditions are close to zero, yet even a slight deviation of validation conditions from calibration conditions causes increases in model errors. Similar decreases in performance have also been reported in other studies, where machine‐learning approaches were calibrated and validated in different environments (Higgins et al. 2020; Luedeling et al. 2021). Asse et al. (2020) also observed that correlative models performed better than process‐oriented approaches when applied to the calibration data, but that process‐based models showed higher performance on new data. On the other hand, we observed that for conditions far outside the calibration conditions, the performance of the machine‐learning model declined less steeply compared to the process‐based model. Machine‐learning models generally rely on large amounts of data and feature uninformed prediction mechanisms that search autonomously for patterns in the training data. These patterns may not represent the system's underlying biological mechanisms, for example, those that govern bud dormancy, making it difficult to capture complex phenomena such as the opposing effects of warming during the two dormancy phases (Arora et al. 2003; Chuine et al. 2016). Several studies have highlighted the poor performance of linear modeling approaches in future projections (Asse et al. 2020; Morin et al. 2009, 2010), even though such approaches may constitute reasonable approximation of temperature responses under current conditions.

Model applications using regime‐clustered groups help separate the effects of data coverage from structural model constraints. This indicates that the observed validity domains largely arise from the models' intrinsic behavior rather than from data insufficiency. While larger and more diverse calibration sets improved performance compared to single‐location datasets, their application did not change the characteristic validity domain patterns of the approaches. The pooled global baseline of the process‐based model performed strongly under most conditions due to maximal data coverage. However, even this global calibration showed reduced accuracy under warm conditions, highlighting persistent structural limitations when additional unmodelled factors become influential. In practical modeling workflows, the diversity of calibration data is a key factor in transferability. It is more beneficial to assemble calibration sets that span a broad range of environmental conditions than to simply increase the volume of similar data. In contrast, the erratic patterns in validation performance of the machine‐learning approach across locations and regime groups suggest that the data structures internalized during calibration often do not match those encountered under new conditions. This further supports the limited extrapolation capacity of machine‐learning models, which tend to rely more on statistical patterns than on underlying processes. A more advanced clustering strategy that jointly considers winter chill and spring forcing could preserve biologically meaningful variation more effectively than single‐axis temperature regimes. Although exploring such multidimensional thermal archetypes is beyond the scope of this study, doing so would be a promising way to refine regime‐based baselines and further disentangle the effects of model structure.

To investigate whether performance decreases for the process‐based model when current models are transferred to conditions that are similar to simulated future scenarios, we compared performance for a range of conditions with simulated scenarios (Figure 4). We generated weather data for periods around two time points (2050, 2085) and three shared socioeconomic pathways (RCP 1.26, 2.45, and 5.85) for each location, relative to a baseline representing conditions in 2000. For these seasons, flowering dates are already known and can be used for model validation. The investigation shows that considerable errors may be incurred when applying models to warmer future scenarios. The application of models calibrated under cold to medium conditions (6°C–13°C) is less error‐prone, however, because these models show broader validity domains. For models calibrated under warm (13°C–15°C) and marginal conditions (15°C–17°C), the temperature range no longer fully covers the conditions spanned by future scenarios, so that model performance cannot be estimated based on the available observation data. However, it seems likely that performance will continue declining with continued increases in temperature. This implies a possibility of escalating errors when applying models to simulated future data. One strategy to deal with this decline in performance could be to include additional data in the calibration process. Such additional information must represent conditions similar to conditions predicted for the coming decades, also known as climate analogue locations (Ramírez Villegas et al. 2011). Models generated from calibration sets that are bolstered with data from such climate analogue locations may be able to predict future trends more reliably (Luedeling 2012).

**FIGURE 5:**
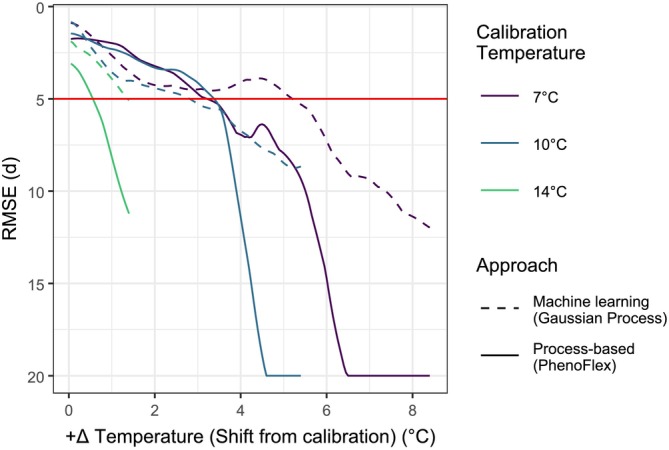
Comparison of model performance along a gradient of environmental conditions depending on different calibration conditions (line colors) and model approaches (line types), quantified by the Root Mean Square Error (RMSE) during validation. The red horizontal line indicates the validity domain threshold with an RMSE of 5 days. The three calibration temperatures cover conditions along the whole gradient. Shorter lines indicate that no warmer data were available to extrapolate the models further. The plot only shows performance for positive temperature shifts.

Model transferability studies evaluate how well models perform when applied to conditions different from those used for their training or calibration (Asse et al. 2020; Yates et al. 2018). They usually assume that deviations between training and calibration conditions come along with a decrease in model transferability (Yates et al. 2018) or shortening forecast horizons (Petchey et al. 2015). Our results support these expectations, but also show that the decrease in performance depends on the initial calibration conditions. Especially for the process‐based approach, these calibration conditions determine if models are transferable to particular future temperature scenarios, as shown for most models calibrated under cold conditions, or whether they cannot be expected to deliver accurate predictions. Comparing the validity domains of both approaches allows selecting modeling strategies that are tailored to specific forecasting intentions and periods of interest (Figure 5). The wide and continuous gradient of conditions covered by our dataset allowed systematic evaluation of how validity domains depend on calibration conditions. This indicates where chill‐limited tipping points, together with structural limitations of the models, begin to negatively affect transferability along the continuous temperature gradient.

A universal calibration that captures plant responses across the entire temperature gradient would be ideal but is rarely achievable in practice because most datasets offer only limited environmental diversity and the collection of phenological observations is usually constrained by the annual cycle. The Japanese dataset is exceptional in this respect, spanning a wide latitudinal range and more than six decades of observations. This provides many climate analogues for future scenarios and supports broader model transferability than is typically achievable. We do not recommend relying on location‐specific models. Instead, we advise merging observations across contrasting conditions to maximize climatic diversity within the calibration set, which improves transferability, particularly when models are intended for use in changing environments. When assembling such datasets, priority should be given to covering a broad span of climate conditions. Data from multiple sites or sources can be combined, if observation protocols are comparable.

Continuous validity domains can be difficult to uncover, but they provide critical insights for transferability beyond the calibration conditions. Our analysis shows that the quality of predictions for validation data depends strongly on the initial calibration temperatures and also on the modeling approach that is chosen. This indicates that comprehensive and systematic cross‐validations are important for robust validation of modeling strategies, especially when applied to future scenarios, which usually differ from the original calibration conditions. This extended validation concept is not limited to phenology data but also applies to other situations where models are used to extrapolate beyond observed conditions. Future experiments could be designed to systematically generate data that can be used for such comprehensive validation. In some cases, it may be possible to combine existing datasets collected under different conditions along relevant gradients to delineate validity domains. Understanding the true validity of models in different domains will enhance our ability to produce accurate forecasts.

## Author Contributions

**Julian N. Bauer:** conceptualization, methodology, writing – original draft, writing – review and editing, visualization. **Katja Schiffers:** conceptualization, methodology, writing – review and editing. **Lars Caspersen:** formal analysis, methodology, writing – review and editing. **Hisayo Yamane:** writing – review and editing, supervision. **Eike Luedeling:** conceptualization, methodology, software, supervision, writing – review and editing.

## Conflicts of Interest

The authors declare no conflicts of interest.

## Supporting information


**Data S1:** gcb70776‐sup‐0001‐Supinfo.zip.

## Data Availability

Phenological data for Somei‐Yoshino cherry blossom (full bloom) are based on observations published by the Japan Meteorological Agency (JMA), meteorological input data were obtained from the NOAA Global Surface Summary of the Day (GSOD) dataset (NOAA National Centers of Environmental Information 1999), and climate projection data were taken from the CMIP6 climate projections provided through the Copernicus Climate Data Store (Copernicus Climate Change Service 2021). Derived datasets, model parameter sets, and all analysis scripts generated in this study are available in this public repository (Bauer et al. 2026).
