# Supplementary material for: Rethinking Model Transferability: Validity Domains as a New Approach to Delineate the Limits of Bloom Date Projections
Source: Glob Chang Biol. 2026 Mar 11;32(3):e70776. doi: 10.1111/gcb.70776 (PMC12976982; doi:10.1111/gcb.70776)
Supplement: Supplementary file 1 — Data S1: gcb70776‐sup‐0001‐Supinfo.zip. [file GCB-32-e70776-s001.zip › gcb70776-sup-0004-TableS1@Bauer_Validity_Domains_Global_Change_SI.docx]

**Supporting Information for**

Rethinking model transferability: Validity domains as a new approach to delineate the limits of bloom date projections

Julian Bauer^1,2^, Katja Schiffers^1^, Lars Caspersen^1^, Hisayo Yamane^2,^ Eike Luedeling^1^

^1^ Institute of Crop Science and Resource Conservation (INRES), University of Bonn

^2^ Graduate School of Agriculture, Kyoto University, Kyoto 606-8502, Japan

Table S1. All General Circulation Models (GCMs) used in this study for each climate scenario described by the Share Socioeconomic Pathway (SSP). The historical simulations for each GCM for the period 1985 – 2014 were downloaded as well.

| GCM | SSP | Citation |
| --- | --- | --- |
| ACCESS-CM2 | ssp126, ssp245, ssp585 | Dix, Martin et al. (2019). CSIRO-ARCCSS ACCESS-CM2 model output prepared for CMIP6 CMIP. Earth System Grid Federation. doi:https://doi.org/10.22033/ESGF/CMIP6.2281 |
| AWI-CM-1-1-MR | ssp126, ssp245, ssp585 | Semmler, Tido et al. (2018). AWI AWI-CM1.1MR model output prepared for CMIP6 CMIP. Earth System Grid Federation. doi:https://doi.org/10.22033/ESGF/CMIP6.359 |
| CIESM | ssp585 | Huang, Wenyu (2019). THU CIESM model output prepared for CMIP6 CMIP. Earth System Grid Federation. doi:https://doi.org/10.22033/ESGF/CMIP6.1352 |
| CMCC-ESM2 | ssp126, ssp245, ssp585 | Lovato, Tomas et al. (2021). CMCC CMCC-ESM2 model output prepared for CMIP6 CMIP. Earth System Grid Federation. doi:https://doi.org/10.22033/ESGF/CMIP6.13164 |
| CNRM-CM6-1 | ssp126 | Voldoire, Aurore (2018). CNRM-CERFACS CNRM-CM6-1 model output prepared for CMIP6 CMIP. Earth System Grid Federation. doi:https://doi.org/10.22033/ESGF/CMIP6.1375 |
| CNRM-CM6-1-HR | ssp126, ssp245, ssp585 | Voldoire, Aurore (2019). CNRM-CERFACS CNRM-CM6-1-HR model output prepared for CMIP6 CMIP. Earth System Grid Federation. doi:https://doi.org/10.22033/ESGF/CMIP6.1385 |
| CNRM-ESM2-1 | ssp126, ssp245, ssp585 | Seferian, Roland (2018). CNRM-CERFACS CNRM-ESM2-1 model output prepared for CMIP6 CMIP. Earth System Grid Federation. doi:https://doi.org/10.22033/ESGF/CMIP6.1391 |
| CanESM5 | ssp126 | Swart, Neil Cameron et al. (2019). CCCma CanESM5 model output prepared for CMIP6 CMIP. Earth System Grid Federation. doi:https://doi.org/10.22033/ESGF/CMIP6.1303 |
| EC-Earth3-CC | ssp245, ssp585 | EC-Earth Consortium (EC-Earth) (2020). EC-Earth-Consortium EC-Earth-3-CC model output prepared for CMIP6 CMIP. Earth System Grid Federation. doi:https://doi.org/10.22033/ESGF/CMIP6.640 |
| EC-Earth3-Veg-LR | ssp126, ssp245, ssp585 | EC-Earth Consortium (EC-Earth) (2020). EC-Earth-Consortium EC-Earth3-Veg-LR model output prepared for CMIP6 CMIP. Earth System Grid Federation. doi:https://doi.org/10.22033/ESGF/CMIP6.643 |
| FGOALS-g3 | ssp126, ssp245, ssp585 | Li, Lijuan (2019). CAS FGOALS-g3 model output prepared for CMIP6 CMIP. Earth System Grid Federation. doi:https://doi.org/10.22033/ESGF/CMIP6.1783 |
| FIO-ESM-2-0 | ssp126, ssp245, ssp585 | Song, Zhenya et al. (2019). FIO-QLNM FIO-ESM2.0 model output prepared for CMIP6 CMIP. Earth System Grid Federation. doi:https://doi.org/10.22033/ESGF/CMIP6.9047 |
| GFDL-ESM4 | ssp126, ssp245, ssp585 | Krasting, John P. et al. (2018). NOAA-GFDL GFDL-ESM4 model output prepared for CMIP6 CMIP. Earth System Grid Federation. doi:https://doi.org/10.22033/ESGF/CMIP6.1407 |
| INM-CM4-8 | ssp126, ssp245, ssp585 | Volodin, Evgeny et al. (2019). INM INM-CM4-8 model output prepared for CMIP6 CMIP. Earth System Grid Federation. doi:https://doi.org/10.22033/ESGF/CMIP6.1422 |
| INM-CM5-0 | ssp126, ssp245, ssp585 | Volodin, Evgeny et al. (2019). INM INM-CM5-0 model output prepared for CMIP6 CMIP. Earth System Grid Federation. doi:https://doi.org/10.22033/ESGF/CMIP6.1423 |
| IPSL-CM6A-LR | ssp126, ssp245, ssp585 | Boucher, Olivier et al. (2018). IPSL IPSL-CM6A-LR model output prepared for CMIP6 CMIP. Earth System Grid Federation. doi:https://doi.org/10.22033/ESGF/CMIP6.1534 |
| MIROC-ES2L | ssp126, ssp245, ssp585 | Hajima, Tomohiro et al. (2019). MIROC MIROC-ES2L model output prepared for CMIP6 CMIP. Earth System Grid Federation. doi:https://doi.org/10.22033/ESGF/CMIP6.902 |
| MIROC6 | ssp126, ssp245, ssp585 | Tatebe, Hiroaki; Watanabe, Masahiro (2018). MIROC MIROC6 model output prepared for CMIP6 CMIP. Earth System Grid Federation. doi:https://doi.org/10.22033/ESGF/CMIP6.881 |
| MPI-ESM1-2-LR | ssp126, ssp245, ssp585 | Wieners, Karl-Hermann et al. (2019). MPI-M MPIESM1.2-LR model output prepared for CMIP6 CMIP. Earth System Grid Federation. doi:https://doi.org/10.22033/ESGF/CMIP6.742 |
| MRI-ESM2-0 | ssp126, ssp245, ssp585 | Yukimoto, Seiji et al. (2019). MRI MRI-ESM2.0 model output prepared for CMIP6 CMIP. Earth System Grid Federation. doi:https://doi.org/10.22033/ESGF/CMIP6.621 |
| NESM3 | ssp126, ssp245, ssp585 | Cao, Jian; Wang, Bin (2019). NUIST NESMv3 model output prepared for CMIP6 CMIP. Earth System Grid Federation. doi:https://doi.org/10.22033/ESGF/CMIP6.2021 |
