# Supplementary material for: Rethinking Model Transferability: Validity Domains as a New Approach to Delineate the Limits of Bloom Date Projections
Source: Glob Chang Biol. 2026 Mar 11;32(3):e70776. doi: 10.1111/gcb.70776 (PMC12976982; doi:10.1111/gcb.70776)
Supplement: Supplementary file 1 — Data S1: gcb70776‐sup‐0001‐Supinfo.zip. [file GCB-32-e70776-s001.zip › gcb70776-sup-0001-TableS1-S2-FigureS1-S2@Bauer_Validity_Domains_GCB_Supporting_Information_final.pdf]

Supporting Information for

Rethinking model transferability: Validity domains as a new approach to delineate the limits of bloom date projections

Julian Bauer[1,2], Katja Schiffers[1], Lars Caspersen[1], Hisayo Yamane[2,] Eike Luedeling[1]
[1] Institute of Crop Science and Resource Conservation (INRES), University of Bonn, Germany
[2] Graduate School of Agriculture, Kyoto University, Kyoto 606-8502, Japan

Table S1. Overview listing all available seasons with phenological data.

Location	Available seasons	No. of years
Akita	1954:1964, 1974:2004, 2007:2022	58
Aomori	1974:2004, 2007:2022	47
Choshi	1954:1964, 1974:2022	60
Fukui	1992:2001, 2003:2004, 2007:2020, 2022	27
Fukuoka	1954:1960, 1962:1964, 1975:2004, 2007:2022	56
Fukushima	1992:2001, 2003:2004, 2007:2020, 2022	27
Gifu	1954:1964, 1992:2001, 2003:2004, 2007:2020, 2022	38
Hakodate	1954:1960, 1962:1964, 1966:1972, 1974:2004, 2007:2022	64
Hikone	1954:1964, 1994:2001, 2003:2004, 2007:2020, 2022	36
Hiroshima	1954:1955, 1962:1964, 1974:1975, 1977:1984, 1986:1987, 1989:2001, 2003:2004, 2007:2022	48
Kagoshima	1954:1964, 1974:2004, 2007:2022	58
Kanazawa	1974:2004, 2007:2022	47
Kobe	1954:1964, 1994:2001, 2003:2004, 2007:2020, 2022	36
Kochi	1955:1957, 1959:1963, 1994:2001, 2003:2004, 2007:2022	34
Kofu	1954:1960, 1962:1964, 1975, 1977:1984, 1986:1987, 1989:2001, 2003:2004, 2007:2020, 2022	51
Kumagaya	1954:1964, 1992:2001, 2003:2004, 2007:2020, 2022	38
Kumamoto	1954:1964, 1974:1975, 1977:1984, 1986:1987, 1989:2001, 2003:2020, 2022	55
Kyoto	1954:1964, 1994:2001, 2003:2004, 2007:2020, 2022	36
Maebashi	1954:1960, 1962:1964, 1974:2004, 2007:2022	57
Matsue	1954:1958, 1964, 1975:2004, 2007:2022	52
Matsuyama	1954:1964, 1974:2022	60
Mito	1954:1964, 1992:2001, 2003:2004, 2007:2022	39
Miyazaki	1974:2022	49
Morioka	1954:1964, 1967:1968, 1974:1975, 1977:1984, 1986:1987, 1989:2001, 2003:2004, 2007:2022	56
Muroran	1954:1964, 1992:2001, 2003:2004, 2007:2020, 2022	38
Nagano	1992:2001, 2003:2004, 2007:2022	28
Nagasaki	1954:1964, 1967:1969, 1972, 1974:2004, 2007:2022	62
Nagoya	1954:1958, 1960:1964, 1966:1972, 1974:2004, 2007:2022	64
Nara	1954:1964, 1994:2001, 2003:2004, 2007:2020, 2022	36
Niigata	1974:1984, 1986:1987, 1989:2001, 2003:2004, 2007:2022	44
Oita	1954:1964, 1974:2022	60
Okayama	1954:1964, 1994:2001, 2003:2004, 2007:2020, 2022	36
Osaka	1957, 1959:1960, 1962, 1974:2004, 2007:2022	51
Saga	1954:1964, 1993:2001, 2003:2004, 2007:2020, 2022	37
Sapporo	1954:1957, 1959:1964, 1966:1972, 1974:2004, 2007:2022	64
Sendai	1974:1982, 1984:2004, 2007:2022	46

Shimonoseki	1954:1964, 1974:1975, 1977:1984, 1986:1987, 1989:2001, 2003:2004, 2007:2020, 2022	53
Shizuoka	1954:1961, 1964, 1992:2001, 2003:2020, 2022	38
Takamatsu	1954:1955, 1962:1964, 1974:2004, 2007:2022	52
Tokushima	1954:1964, 1994:2001, 2003:2004, 2007:2022	37
Tokyo	1955:1964, 1966:1972, 1974:2004, 2007:2022	64
Tottori	1954:1964, 1975:2004, 2007:2022	57
Toyama	1992:2001, 2003:2004, 2007:2020, 2022	27
Tsu	1954:1964, 1992:2001, 2003:2004, 2007:2022	39
Utsunomiya	1992:2001, 2003:2004, 2007:2020, 2022	27
Wakayama	1955:1964, 1994:2001, 2003:2004, 2007:2020, 2022	35
Yamagata	1954:1964, 1992:2001, 2003:2004, 2007:2020, 2022	38
Yokohama	1992:2001, 2003:2004, 2007:2020, 2022	27

Table S2. All General Circulation Models (GCMs) used in this study for each climate scenario described by the Share Socioeconomic Pathway (SSP). The historical simulations for each GCM for the period 1985 – 2014 were downloaded as well.

GCM	SSP	Citation
ACCESS-CM2	ssp126, ssp245, ssp585	Dix, Martin et al. (2019). CSIRO-ARCCSS ACCESS-CM2 model output prepared for CMIP6 CMIP. Earth System Grid Federation. doi:https://doi.org/10.22033/ESGF/CMIP6.2281
AWI-CM-1-1-MR	ssp126, ssp245, ssp585	Semmler, Tido et al. (2018). AWI AWI-CM1.1MR model output prepared for CMIP6 CMIP. Earth System Grid Federation. doi:https://doi.org/10.22033/ESGF/CMIP6.359
CIESM	ssp585	Huang, Wenyu (2019). THU CIESM model output prepared for CMIP6 CMIP. Earth System Grid Federation. doi:https://doi.org/10.22033/ESGF/CMIP6.1352
CMCC-ESM2	ssp126, ssp245, ssp585	Lovato, Tomas et al. (2021). CMCC CMCC-ESM2 model output prepared for CMIP6 CMIP. Earth System Grid Federation. doi:https://doi.org/10.22033/ESGF/CMIP6.13164
CNRM-CM6-1	ssp126	Voldoire, Aurore (2018). CNRM-CERFACS CNRM-CM6-1 model output prepared for CMIP6 CMIP. Earth System Grid Federation. doi:https://doi.org/10.22033/ESGF/CMIP6.1375
CNRM-CM6-1-HR	ssp126, ssp245, ssp585	Voldoire, Aurore (2019). CNRM-CERFACS CNRM-CM6-1-HR model output prepared for CMIP6 CMIP. Earth System Grid Federation. doi:https://doi.org/10.22033/ESGF/CMIP6.1385
CNRM-ESM2-1	ssp126, ssp245, ssp585	Seferian, Roland (2018). CNRM-CERFACS CNRM-ESM2-1 model output prepared for CMIP6 CMIP. Earth System Grid Federation. doi:https://doi.org/10.22033/ESGF/CMIP6.1391
CanESM5	ssp126	Swart, Neil Cameron et al. (2019). CCCma CanESM5 model output prepared for CMIP6 CMIP. Earth System Grid Federation. doi:https://doi.org/10.22033/ESGF/CMIP6.1303
EC-Earth3-CC	ssp245, ssp585	EC-Earth Consortium (EC-Earth) (2020). EC-Earth-Consortium EC-Earth-3-CC model output prepared for CMIP6 CMIP. Earth System Grid Federation. doi:https://doi.org/10.22033/ESGF/CMIP6.640
EC-Earth3-Veg-LR	ssp126, ssp245, ssp585	EC-Earth Consortium (EC-Earth) (2020). EC-Earth-Consortium EC-Earth3-Veg-LR model output prepared for CMIP6 CMIP. Earth System Grid Federation. doi:https://doi.org/10.22033/ESGF/CMIP6.643
FGOALS-g3	ssp126, ssp245, ssp585	Li, Lijuan (2019). CAS FGOALS-g3 model output prepared for CMIP6 CMIP. Earth System Grid Federation. doi:https://doi.org/10.22033/ESGF/CMIP6.1783

FIO-ESM-2-0	ssp126, ssp245, ssp585	Song, Zhenya et al. (2019). FIO-QLNM FIO-ESM2.0 model output prepared for CMIP6 CMIP. Earth System Grid Federation. doi:https://doi.org/10.22033/ESGF/CMIP6.9047
GFDL-ESM4	ssp126, ssp245, ssp585	Krasting, John P. et al. (2018). NOAA-GFDL GFDL-ESM4 model output prepared for CMIP6 CMIP. Earth System Grid Federation. doi:https://doi.org/10.22033/ESGF/CMIP6.1407
INM-CM4-8	ssp126, ssp245, ssp585	Volodin, Evgeny et al. (2019). INM INM-CM4-8 model output prepared for CMIP6 CMIP. Earth System Grid Federation. doi:https://doi.org/10.22033/ESGF/CMIP6.1422
INM-CM5-0	ssp126, ssp245, ssp585	Volodin, Evgeny et al. (2019). INM INM-CM5-0 model output prepared for CMIP6 CMIP. Earth System Grid Federation. doi:https://doi.org/10.22033/ESGF/CMIP6.1423
IPSL-CM6A-LR	ssp126, ssp245, ssp585	Boucher, Olivier et al. (2018). IPSL IPSL-CM6A-LR model output prepared for CMIP6 CMIP. Earth System Grid Federation. doi:https://doi.org/10.22033/ESGF/CMIP6.1534
MIROC-ES2L	ssp126, ssp245, ssp585	Hajima, Tomohiro et al. (2019). MIROC MIROC-ES2L model output prepared for CMIP6 CMIP. Earth System Grid Federation. doi:https://doi.org/10.22033/ESGF/CMIP6.902
MIROC6	ssp126, ssp245, ssp585	Tatebe, Hiroaki; Watanabe, Masahiro (2018). MIROC MIROC6 model output prepared for CMIP6 CMIP. Earth System Grid Federation. doi:https://doi.org/10.22033/ESGF/CMIP6.881
MPI-ESM1-2-LR	ssp126, ssp245, ssp585	Wieners, Karl-Hermann et al. (2019). MPI-M MPIESM1.2-LR model output prepared for CMIP6 CMIP. Earth System Grid Federation. doi:https://doi.org/10.22033/ESGF/CMIP6.742
MRI-ESM2-0	ssp126, ssp245, ssp585	Yukimoto, Seiji et al. (2019). MRI MRI-ESM2.0 model output prepared for CMIP6 CMIP. Earth System Grid Federation. doi:https://doi.org/10.22033/ESGF/CMIP6.621
NESM3	ssp126, ssp245, ssp585	Cao, Jian; Wang, Bin (2019). NUIST NESMv3 model output prepared for CMIP6 CMIP. Earth System Grid Federation. doi:https://doi.org/10.22033/ESGF/CMIP6.2021

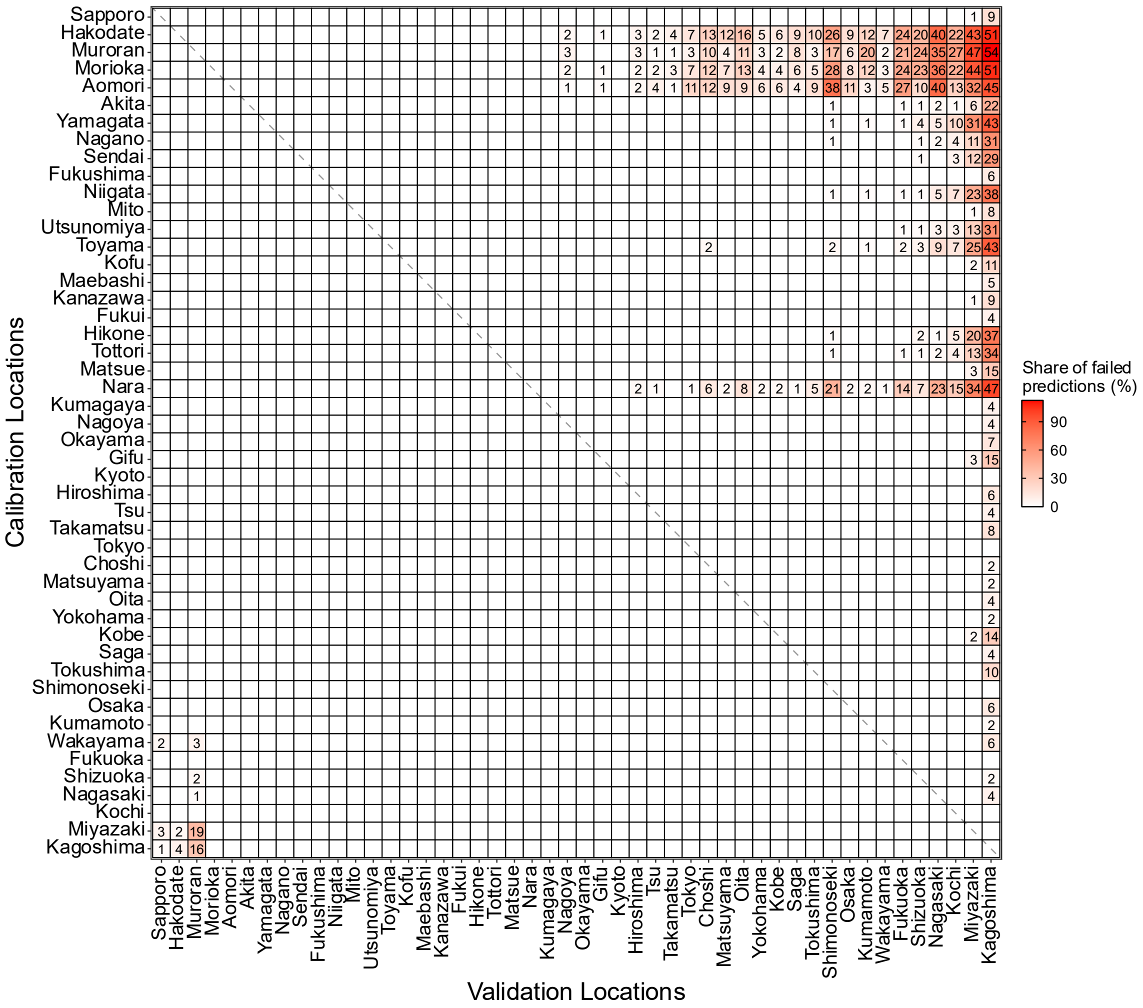

Figure S1. Share of failed/non-computable predictions (as color) and amount of failed predictions (as text) for all combinations of calibration and validation locations for the process-based approach.

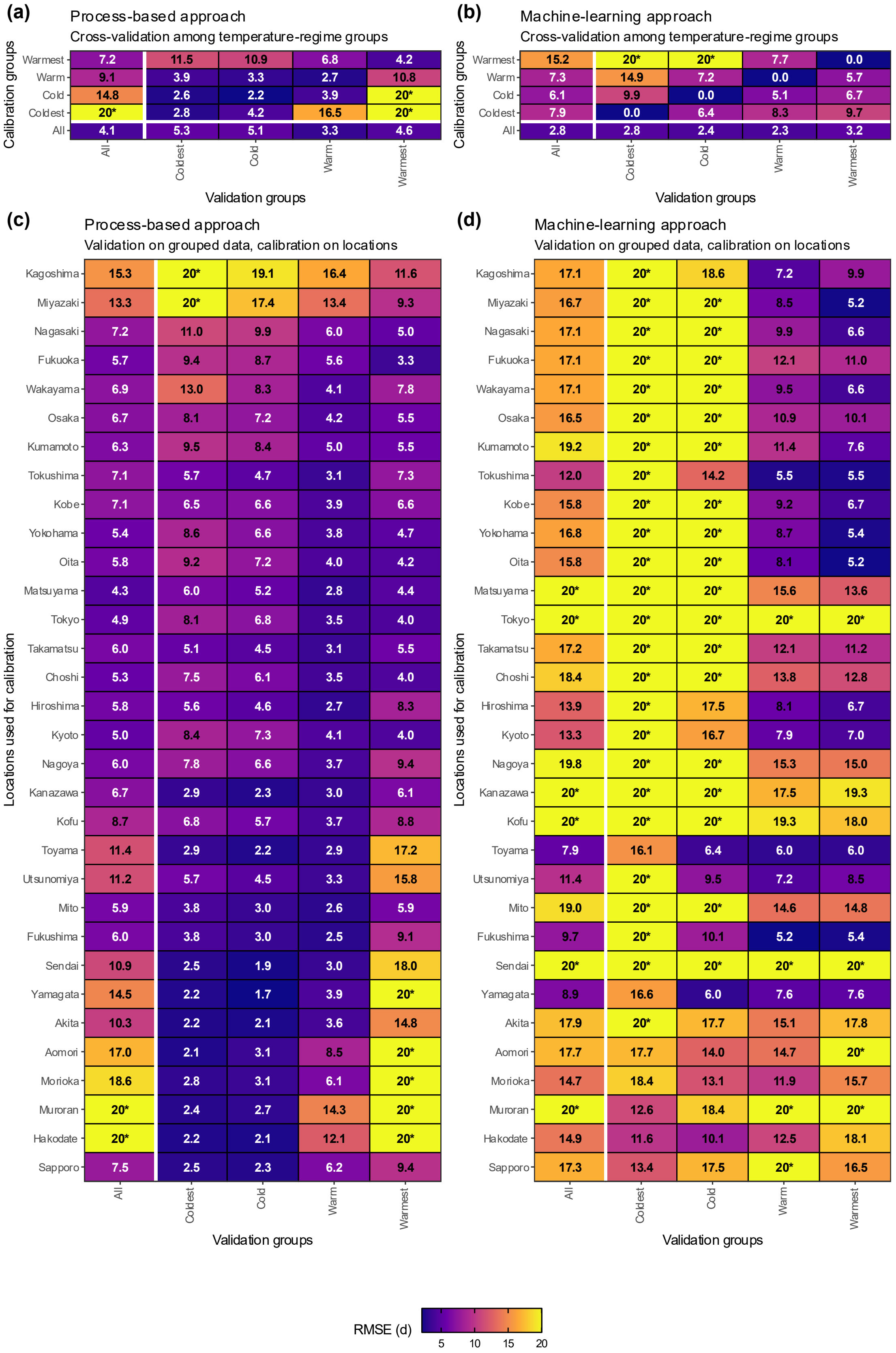
(a)
Process-based approach
Cross-validation among temperature-regime groups
Calibration groups
Warmest 7.2 11.5 10.9 6.8 4.2
Warm 9.1 3.9 3.3 2.7 10.8
Cold 14.8 2.6 2.2 3.9 20*
Coldest 20* 2.8 4.2 16.5 20*
All 4.1 5.3 5.1 3.3 4.6
All Coldest Cold Warm Warmest
Validation groups
(b)
Machine-learning approach
Cross-validation among temperature-regime groups
Calibration groups
Warmest 15.2 20* 20* 7.7 0.0
Warm 7.3 14.9 7.2 0.0 5.7
Cold 6.1 9.9 0.0 5.1 6.7
Coldest 7.9 0.0 6.4 8.3 9.7
All 2.8 2.8 2.4 2.3 3.2
All Coldest Cold Warm Warmest
Validation groups
(c)
Process-based approach
Validation on grouped data, calibration on locations
Locations used for calibration
Kagoshima 15.3 20* 19.1 16.4 11.6
Miyazaki 13.3 20* 17.4 13.4 9.3
Nagasaki 7.2 11.0 9.9 6.0 5.0
Fukuoka 5.7 9.4 8.7 5.6 3.3
Wakayama 6.9 13.0 8.3 4.1 7.8
Osaka 6.7 8.1 7.2 4.2 5.5
Kumamoto 6.3 9.5 8.4 5.0 5.5
Tokushima 7.1 5.7 4.7 3.1 7.3
Kobe 7.1 6.5 6.6 3.9 6.6
Yokohama 5.4 8.6 6.6 3.8 4.7
Oita 5.8 9.2 7.2 4.0 4.2
Matsuyama 4.3 6.0 5.2 2.8 4.4
Tokyo 4.9 8.1 6.8 3.5 4.0
Takamatsu 6.0 5.1 4.5 3.1 5.5
Choshi 5.3 7.5 6.1 3.5 4.0
Hiroshima 5.8 5.6 4.6 2.7 8.3
Kyoto 5.0 8.4 7.3 4.1 4.0
Nagoya 6.0 7.8 6.6 3.7 9.4
Kanazawa 6.7 2.9 2.3 3.0 6.1
Kofu 8.7 6.8 5.7 3.7 8.8
Toyama 11.4 2.9 2.2 2.9 17.2
Utsunomiya 11.2 5.7 4.5 3.3 15.8
Mito 5.9 3.8 3.0 2.6 5.9
Fukushima 6.0 3.8 3.0 2.5 9.1
Sendai 10.9 2.5 1.9 3.0 18.0
Yamagata 14.5 2.2 1.7 3.9 20*
Akita 10.3 2.2 2.1 3.6 14.8
Aomori 17.0 2.1 3.1 8.5 20*
Morioka 18.6 2.8 3.1 6.1 20*
Muroran 20* 2.4 2.7 14.3 20*
Hakodate 20* 2.2 2.1 12.1 20*
Sapporo 7.5 2.5 2.3 6.2 9.4
All Coldest Cold Warm Warmest
Validation groups
(d)
Machine-learning approach
Validation on grouped data, calibration on locations
Locations used for calibration
Kagoshima 17.1 20* 18.6 7.2 9.9
Miyazaki 16.7 20* 20* 8.5 5.2
Nagasaki 17.1 20* 20* 9.9 6.6
Fukuoka 17.1 20* 20* 12.1 11.0
Wakayama 17.1 20* 20* 9.5 6.6
Osaka 16.5 20* 20* 10.9 10.1
Kumamoto 19.2 20* 20* 11.4 7.6
Tokushima 12.0 20* 14.2 5.5 5.5
Kobe 15.8 20* 20* 9.2 6.7
Yokohama 16.8 20* 20* 8.7 5.4
Oita 15.8 20* 20* 8.1 5.2
Matsuyama 20* 20* 20* 15.6 13.6
Tokyo 20* 20* 20* 20* 20*
Takamatsu 17.2 20* 20* 12.1 11.2
Choshi 18.4 20* 20* 13.8 12.8
Hiroshima 13.9 20* 17.5 8.1 6.7
Kyoto 13.3 20* 16.7 7.9 7.0
Nagoya 19.8 20* 20* 15.3 15.0
Kanazawa 20* 20* 20* 17.5 19.3
Kofu 20* 20* 20* 19.3 18.0
Toyama 7.9 16.1 6.4 6.0 6.0
Utsunomiya 11.4 20* 9.5 7.2 8.5
Mito 19.0 20* 20* 14.6 14.8
Fukushima 9.7 20* 10.1 5.2 5.4
Sendai 20* 20* 20* 20* 20*
Yamagata 8.9 16.6 6.0 7.6 7.6
Akita 17.9 20* 17.7 15.1 17.8
Aomori 17.7 17.7 14.0 14.7 20*
Morioka 14.7 18.4 13.1 11.9 15.7
Muroran 20* 12.6 18.4 20* 20*
Hakodate 14.9 11.6 10.1 12.5 18.1
Sapporo 17.3 13.4 17.5 20* 16.5
All Coldest Cold Warm Warmest
Validation groups
RMSE (d)
5 10 15 20

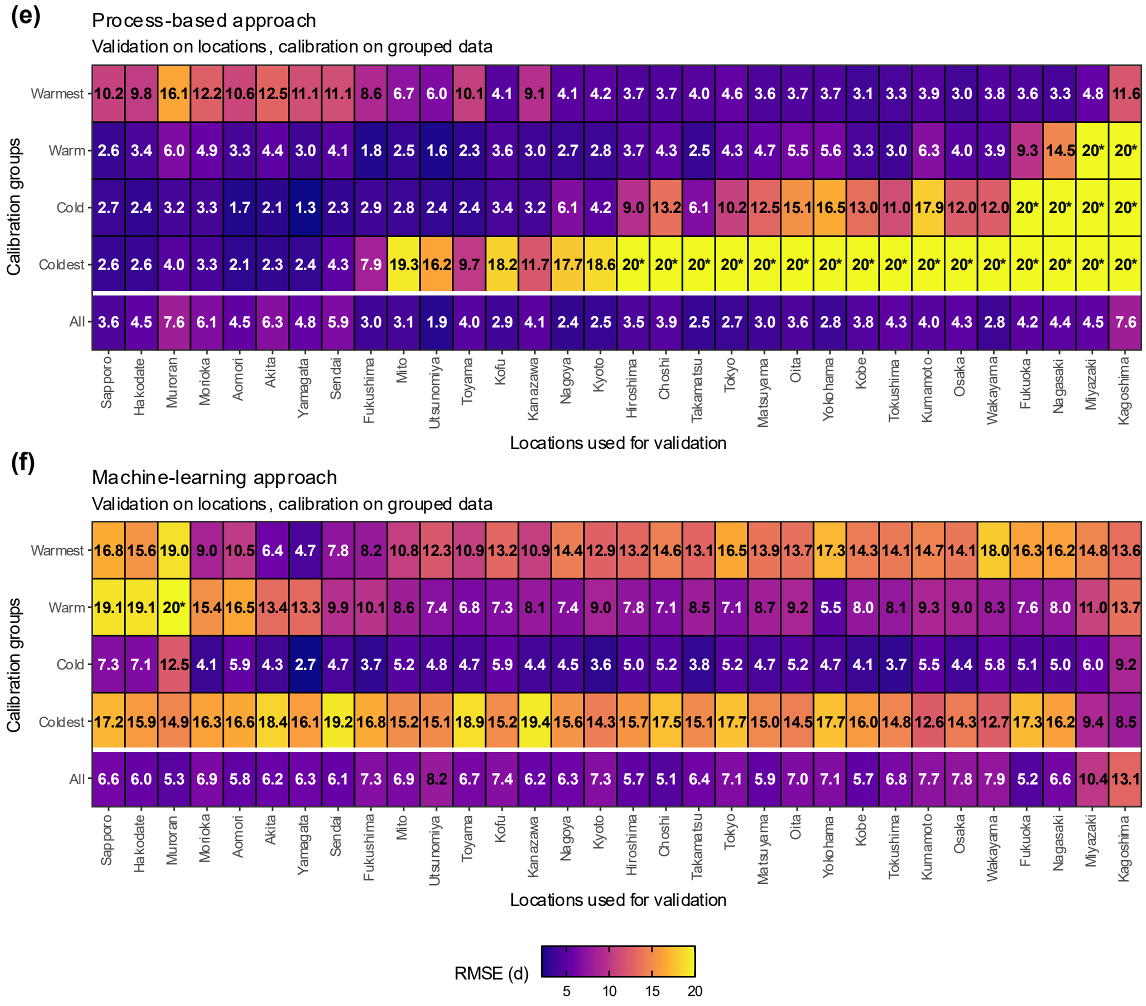

Figure S2. Performance of regime-clustered temperature groups and a pooled global dataset when calibrated and validated across groups and individual locations using the process-based and machine-learning approaches. We divided the full range of seasonal mean temperatures into four regime groups (Coldest: 5.1–7.7 °C; Cold: 7.7–10.3 °C; Warm: 10.3–12.8 °C; Warmest: 12.8–15.4 °C). We then sampled 100 seasons from each group. The pooled global dataset comprised all 2,189 available seasons. We calibrated process-based and machine-learning models for each of the five datasets. Panels (a) (process-based) and (b) (machine-learning) show model performance obtained by cross-validating the four temperature-regime groups and the global set. Panels (c) and (d) show how a selection of 32 location-specific models responded when validated against the same five datasets using the process-based (c) and machine-learning (d) approaches. Panels (e) and (f) present the reverse setting, in which the five temperature regimes provided the calibration data and the individual locations served as validation datasets. All errors greater than 20 days are truncated and marked with an asterisk to avoid distorting the color gradient.
